# miR-6805-5p as a biomarker of cisplatin-induced nephrotoxicity in patients with head and neck cancer

**DOI:** 10.3389/fphar.2023.1275238

**Published:** 2023-11-28

**Authors:** Nadine De Godoy Torso, Julia Coelho França Quintanilha, Maria Aparecida Cursino, Eder De Carvalho Pincinato, Pía Loren, Luis A. Salazar, Carmen Silvia Passos Lima, Patricia Moriel

**Affiliations:** ^1^ School of Medical Sciences, Universidade Estadual de Campinas, Campinas, Brazil; ^2^ Center of Molecular Biology and Pharmacogenetics, Scientific and Technological Bioresource Nucleus, Universidad de La Frontera, Temuco, Chile; ^3^ Faculty of Pharmaceutical Sciences, Universidade Estadual de Campinas, Campinas, Brazil

**Keywords:** cisplatin, nephrotoxicity, acute kidney injury, biomarker, microRNA, miRNA

## Abstract

**Introduction:** The standard treatment for head and neck squamous cell carcinoma (HNSCC) is cisplatin chemoradiotherapy. One of the main treatment adverse reactions is nephrotoxicity, for which there is currently no adequate specific and sensitive biomarker. Thus, this study aimed to evaluate the use of microRNAs (miRNAs) as renal biomarker candidates.

**Methods:** This was a retrospective cohort study. Nephrotoxicity was assessed through blood samples collected before and 5 days (D5) after chemotherapy. MiRNAs were extracted from urine samples collected at baseline and D5, and RNA sequencing identified miRNAs differentially expressed between participants with and without cisplatin-induced nephrotoxicity.

**Results:** A total of 49 participants were included (*n* = 49). A significant difference was seen between the two groups for traditional renal markers (serum creatinine and creatinine clearance) and for the acute kidney injury (AKI) categories. Among the six miRNAs evaluated as biomarkers, four were upregulated (hsa-miR-6729-5p, hsa-miR-1238-5p, hsa-miR-4706, and hsa-miR-4322) and two were downregulated (hsa-miR-6805-5p and hsa-miR-21-5p), but only hsa-miR-6805-5p had a significant difference (*p* < 0.0001). Its receiver operating characteristic curve revealed excellent specificity (0.920) for its expression fluctuation assessment, while its absolute expression in D5 showed greater sensitivity (0.792).

**Conclusion:** So, the integrated use of these two parameters seems to be an interesting approach for AKI.

## 1 Introduction

Head and neck squamous cell carcinoma (HNSCC) is one of the most prevalent cancers worldwide (Sung et al., 2021), arising in the stratified epithelium of the oral cavity, oropharynx, hypopharynx, larynx, or nasopharynx (Chow, 2020). At the time of diagnosis, most patients had locally advanced disease (stages III and IV) (Chow, 2020), for which the standard treatment recommendation is concomitant chemoradiotherapy with high doses of cisplatin (Caudell et al., 2022).

Although cisplatin is a well-known anticancer agent approved for the treatment of tumors of different etiologies (Rottenberg et al., 2021), it is also known to be associated with significant adverse reactions. Among them, nephrotoxicity tends to be the most frequent and the major contributor to chemotherapy dose reduction or delays and cessation of treatment, thereby impacting patients’ survival and disease progression (Duffy et al., 2018). Besides, even if the long-term consequences of cisplatin-induced nephrotoxicity remain unclear (Van Der Vorst et al., 2019), it is well-known that the immediate impact may be acute kidney injury (AKI), a clinical syndrome evidenced by an abrupt decrease in kidney function as a result of injury and death of tubular epithelial cells (Van Der Vorst et al., 2019; McSweeney et al., 2021).

The traditional biomarkers routinely used in the clinic are helpful in monitoring nephrotoxicity; however, they have considerable limitations, whether low sensitivity or specificity, in detecting AKI-associated early events (Waikar et al., 2012; Van Meer et al., 2014; Torso et al., 2023b). For this reason, the last few years have seen an evident search for novel kidney biomarkers.

Because necrotic or apoptotic cell death can culminate in the release of microRNAs (miRNAs) into biofluids (Roderburg and Luedde, 2014; Koturbash et al., 2015), they are among the most promising candidates for renal biomarker development. MiRNAs are a class of non-coding RNAs, approximately 20 bp in length, and essential regulators of gene expression (Krol et al., 2010; Saliminejad et al., 2019). Besides being involved in a variety of cellular processes, whether physiological or pathological (Small and Olson, 2011; Li et al., 2013; Zhang et al., 2017), circulating miRNAs are considered advantageous alternative biomarkers also because of their specific expression pattern in different tissues or pathological states (Fan et al., 2016; Torso et al., 2021), relatively simple laboratory analysis methods, and stability in a variety of body fluids.

Since the first evidence of the role of miRNAs in kidney functions (Kato et al., 2007; Ho et al., 2008), evidence associating miRNAs with AKI has become stronger. Some miRNA specimens were previously associated with apoptosis, necrosis, fibrosis, and inflammatory processes (Fan et al., 2016; Torso et al., 2021), all essential stress responses in the pathogenesis of cisplatin nephrotoxicity (Tang et al., 2023). However, their use as biomarkers in human cohorts has been poorly investigated (Torso et al., 2021; Mahtal et al., 2022). Despite years of intensive research, few advancements have been made regarding the timely detection of cisplatin-induced nephrotoxicity. Thus, this study aimed to validate the use of six miRNAs as noninvasive renal biomarkers in HNSCC patients treated with cisplatin.

## 2 Materials and methods

### 2.1 Study design and ethical considerations

This was a retrospective cohort study. This study was reported in accordance with the STROBE Statement and Checklist for Cohort Studies available from the STROBE website (https://www.strobe-statement.org/). The study was conducted in accordance with the Declaration of Helsinki and was approved by the Ethics Committee of the University of Campinas (protocol code: 65397517.7.0000.5404, 8 February 2021). Informed consent was obtained from all the subjects involved in the study.

### 2.2 Participants and treatment regimen

This study was conducted in the Clinical Oncology Department of the *Hospital de Clínicas*, University of Campinas (HC-UNICAMP), a large teaching hospital in Campinas, Brazil. The inclusion criteria and antineoplastic treatment regimen were described previously (Torso et al., 2023b).

### 2.3 Nephrotoxicity assessment

Blood samples were collected before and 5 days (D5) after the first treatment cycle to assess cisplatin-induced nephrotoxicity; baseline values were then compared to those results after cisplatin administration. Laboratory markers examined for nephrotoxicity were serum creatinine (SCr), estimated creatinine clearance (estimated by the Cockcroft-Gault formula (Cockcroft and Gault, 1976)), urea, sodium, magnesium, calcium, phosphorus, and potassium; variations from baseline values of these markers were graded for severity in accordance with the Common Toxicity Criteria for Adverse Events (CTCAE) version 4 (NCI, NIH, 2009). The Risk, Injury, Failure, Loss, and End-Stage Kidney Disease (RIFLE) (Bellomo et al., 2004) and Acute Kidney Injury Network (AKIN) (Mehta et al., 2007) criteria were also used to categorize AKI.

### 2.4 MiRNA sequencing

For miRNA sequencing, urine samples collected on D5 were used because, according to the research of Pavkovic et al. (2014), maximum changes in miRNAs seem to occur after 5 days of cisplatin administration. After collection, the samples were centrifuged at 2,500 rpm at 4°C for 10 min; the supernatant was aliquoted and stored in a −80°C freezer until analysis. Details of the miRNA sequencing have been previously reported and it was performed according to published methods (Quintanilha et al., 2021). Briefly, massive sequencing was performed to identify the miRNAs differentially expressed between those participants with cisplatin nephrotoxicity and those without cisplatin nephrotoxicity.

#### 2.4.1 *In-silico* analysis of potential targets of miRNAs identified in sequencing

A total of 10 miRNAs were selected for *in silico* analysis: 9 from sequencing (fold regulation>7.0 or < -7.0), and the 10th (hsa-miR-21-5p) was chosen for showing promising results in the literature review (Torso et al., 2021). Initially, the database miRWalk (miRWalk, 2021; Dweep and Gretz, 2015) was used for the identification of potential predicted target genes of miRNAs of interest.

In the next step, a matrix was built with the genes predicted by such miRNAs; only the genes predicted by the 10 miRNAs included in the analysis were selected (since this criterion already resulted in more than 6,000 genes). The result was used to perform an unsupervised enrichment analysis by the software IPA^®^ (Qiagen Bioinformatics), to identify the main canonical signaling pathways in which the differentially expressed miRNAs were involved. Only predicted pathways with a value of *p* ≤ 0.001 were selected for further analysis.

**FIGURE 1:**
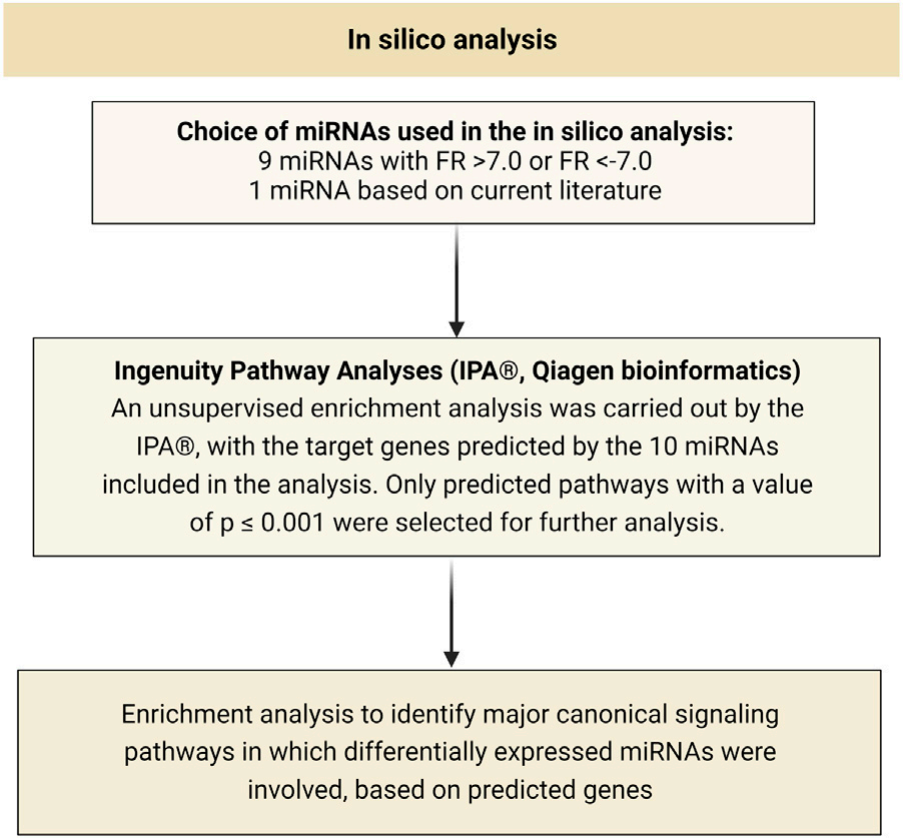
Schematic representation of all steps performed in the *in silico* analysis. FR, fold regulation.

The third stage of the *in silico* analysis was the study of the canonical signaling pathways resulting from the enrichment analysis; this last step was performed again using the IPA^®^. Figure 1 schematically summarizes the implementation of the entire process.

### 2.5 Criteria for selecting possible biomarker candidates

Considering the RNA-Seq expression data, three criteria were used to select the miRNA candidates for biomarkers of nephrotoxicity: (1) the miRNA must have a fold regulation value > 10 or < -10; (2) the miRNA must have a *p*-value < 0,05; and (3) the miRNA must not have comments by the GeneGlobe Data Analysis Center (Qiagen, Germany). In addition to the miRNAs selected from next-generation sequencing, hsa-miR-21-5p was also selected for validation in the cohort due to its previous promising results for diagnosing AKI (Saal and Harvey, 2009).

### 2.6 MiRNA expression assessment

Details of miRNA expression assessment methods were presented previously (Torso et al., 2023a) and are reproduced here.

miRNAs were extracted from urine samples collected at baseline and on D5 using the miRNeasy Serum/Plasma Kit (Qiagen, Germany), following the manufacturer’s instructions. During extraction procedures, 3 × 10^7^ copies of the synthetic miRNA *Caenorhabditis elegans* miR-39 (cel-miR-39) were added to be used as an exogenous control (spike-in). cDNA was synthesized using the TaqMan™ Advanced miRNA cDNA Synthesis Kit (Applied Biosystems, Waltham, MA, United States), following the manufacturer’s instructions.

RT-qPCR reactions were performed on a Rotor-Gene Q (Qiagen, Germany), using TaqMan™ Advanced miRNA Assays (Applied Biosystems, United States) for the six miRNAs selected for validation, as well as for cel-miR-39 (spike-in) (Kroh et al., 2010) and hsa-miR-875-5p (endogenous normalizer) (Torso et al., 2023a). The total reaction volume was reduced to 10 μL, consisting of 5 µL of TaqMan^®^ Fast Advanced Master Mix (2×) (Applied Biosystems, United States), 0.5 µL of TaqMan^®^ Advanced miRNA Assay (20×) (Applied Biosystems, Waltham, MA, United States), 2 µL of RNase-free water, and 2.5 µL of diluted cDNA (1:10). Raw data were evaluated using Rotor-Gene Q Series Software (Qiagen, Germany). As part of the quality control, samples whose cel-miR-39 expression was above two standard deviations were excluded from the analysis.

Expression changes were evaluated using the 2^−ΔΔCT^ method, where ΔCt is the difference between the threshold cycle of the target gene and the endogenous control (hsa-miR-875-5p) (Livak and Schmittgen, 2001; Schmittgen and Livak, 2008), to remove variations that were not related to the biological condition studied. The result is given in fold change, that is, how many times the target miRNA showed increased or decreased expression in the group with nephrotoxicity compared to the group without cisplatin-induced nephrotoxicity.

### 2.7 *In-silico* analysis of hsa-miR-6805-5p

As hsa-miR-6805-5p showed better results as a biomarker of cisplatin-associated nephrotoxicity, an *in silico* analysis was performed to identify predicted target pathways for this miRNA that were potentially associated with nephrotoxicity. The analysis was conducted using the online software mirPath v.3 (https://dianalab.e-ce.uth.gr/html/mirpathv3/index.php?r=mirpath, accessed on 14 June 2023).

### 2.8 Statistical analysis

For the analysis of clinical and demographic data, absolute frequencies/percentages, and measures of position (mean) and dispersion (standard deviation) are presented. Nephrotoxicity markers and participant profiles in the two groups studied were compared at baseline and D5 using the Mann-Whitney test or Fisher’s exact test. Data normality was tested using the Shapiro-Wilk test. In the validation phase, miRNA expressions were compared between groups with and without cisplatin-induced nephrotoxicity at baseline and D5 using the Mann-Whitney test, and ROC curves were generated for comparisons with *p* < 0.05. A value of *p* < 0.05 was considered statistically significant for all analyses. All statistical analyses were performed using GraphPad Prism v.9.1.0 software for Windows (GraphPad Software, Inc., San Diego, California, United States).

## 3 Results

### 3.1 Participants

**FIGURE 2:**
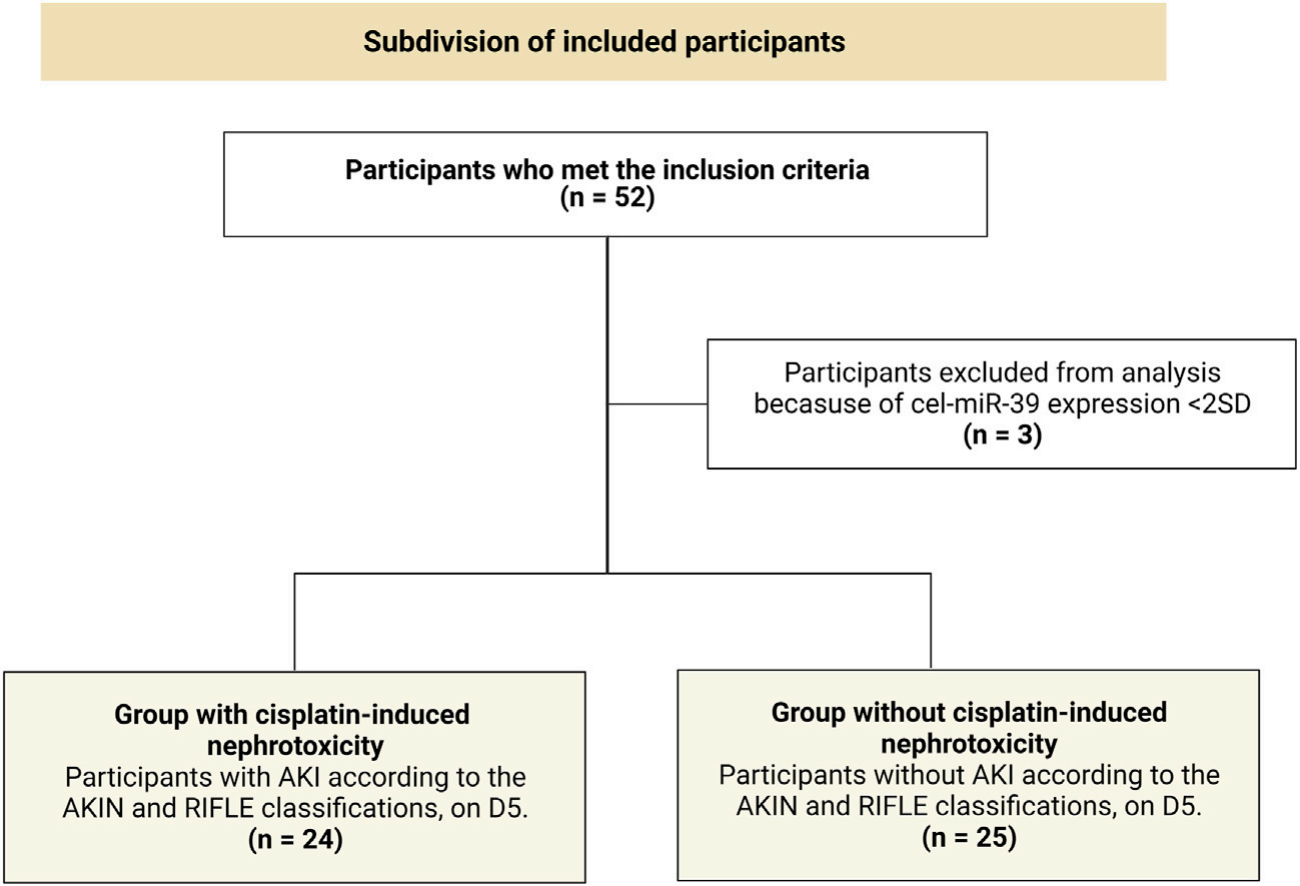
Subdivision of included participants in the present study into two groups. AKIN, Acute Kidney Injury Network; D5, 5th day from the first cisplatin chemotherapy cycle; SD, standard deviation; AKI, acute kidney injury; n, absolute number of participants; RIFLE, Risk, Injury, Failure, Loss, and End-stage kidney disease.

A total of 52 participants met the inclusion criteria for this study, for whom the urinary miRNA profile assessment was performed. Three of them were excluded from the analyses for having cel-miR-39 expression levels above two standard deviations (these data were even re-assessed to confirm the exclusion of these participants). The remaining 49 participants (n = 49) were subdivided into two groups: a group of participants who did not have cisplatin-induced nephrotoxicity (*n* = 25) and a group of participants who did (*n* = 24) (Figure 2).

The clinical characteristics of all 49 participants included in this study are shown in Table 1. Overall, statistical analysis showed that the demographic and clinical characteristics were very similar between the two groups: briefly, the mean age was quite similar, as were the proportions of male and female participants. Most participants were self-identified as white, had a history of smoking and alcohol consumption, and were classified with a Karnofsky Performance Status (KPS) of 90%. As for the disease, participants were mainly diagnosed at an advanced stage (stage IV), with the oral cavity, larynx, and oropharynx as the most frequent tumor sites in the cohort.

**TABLE 1:** Demographic and clinical data of included participants (*n* = 49).

Variable	Participants with cisplatin-induced nephrotoxicity (*n* = 24)	Participants without cisplatin-induced nephrotoxicity (*n* = 25)	*p*-value
**Age at diagnosis** (mean ± SD, years)	58.46 ± 7.26	58.83 ± 6.55	0.7259^a^
**Gender** (*n*, %)			
Male	21 (87.5)	21 (84.0)	1.000^b^
Female	3 (12.5)	4 (16.0)	
**Ethnicity** (*n*, %)			
Caucasian	16 (66.7)	22 (88.0)	0.0736^b^
Non-Caucasian	8 (33.3)	3 (12.0)	
**Smoking** (*n*, %)			
Never smoked	2 (8.3)	2 (8.0)	1.000^b^
Smoker	22 (91.7)	23 (92.0)	
**Alcohol consumption** (*n*, %)			
Abstainer	4 (16.7)	4 (16.0)	1.000^b^
Drinker	20 (83.3)	21 (84.0)	
**Tumor site** (*n*, %)			
Oral cavity	6 (25.0)	9 (36.0)	0.9123^b^
Larynx	6 (25.0)	5 (20.0)	
Hypopharynx	5 (20.8)	4 (16.0)	
Hypopharynx and larynx	0 (0)	1 (4.0)	
Oropharynx	5 (20.8)	6 (24.0)	
Not accessed	2 (8.3)	0 (0)	
**Tumor stage** (*n*, %)			
I	0	0	-
II	3 (12.5)	3 (12.0)	
III	3 (12.5)	2 (8.0)	
IV	18 (75.0)	20 (80.0)	
**KPS** (*n*, %)			
100	4 (16.7)	2 (8.0)	-
90	14 (58.3)	21 (84.0)	
80	5 (20.8)	2 (8.0)	
70	1 (4.2)	0 (0)	
**BMI** (mean ± SD)	23.4 ± 4.8	22.6 ± 4.8	0.6171^a^
**Comorbidities** ^c^ (n, %)			
Hypertension	9 (37.5)	5 (20.0)	—
Diabetes	4 (16.7)	2 (8.0)	

BMI, body mass index; KPS, karnofsky performance status; *n*, absolute number of patients; SD, standard deviation.

^a^ Mann-Whitney test.

^b^ Fisher’s exact test.

^c^ Patients may have two comorbidities simultaneously.

The statistically significant differences were showed by the bold.

### 3.2 Nephrotoxicity assessment

Table 2 shows the nephrotoxicity parameters for both groups of participants. When comparing their renal function 5 days after the first cycle of chemotherapy, a significant difference was observed both for laboratory parameters (alteration in serum creatinine and creatinine clearance) and for RIFLE (Bellomo et al., 2004) and AKIN (Mehta et al., 2007) classifications.

**TABLE 2:** Renal biomarkers, and AKIN and RIFLE classifications for the 49 participants.

	Participants with cisplatin-induced nephrotoxicity (*n* = 24)		Participants without cisplatin-induced nephrotoxicity (*n* = 25)		*p*-value
	Baseline	D5	Baseline	D5	
**SCr (mg/dL) (mean ± SD)**	0.8 ± 0.2	1.9 ± 1.4	0.9 ± 0.2	0.9 ± 0.2	-
**SCr variation (mg/dL) (D5 - Baseline; mean ± SD)**	1.2 ± 1.4		0.1 ± 0.1		**<0.0001** ^a^
**Creatinine clearance (mL/min) (mean ± SD)**	90.6 ± 6.7	44.9 ± 8.2	80.4 ± 21.0	72.7 ± 20.0	-
**Creatinine clearance variation (mL/min) (D5 - Baseline; mean ± SD)**	−45.7 ± 25.7		−7.7 ± 7.7		**<0.0001** ^a^
**CTCAE - Increased SCr (D5) (*n*, %)**					
Grade 0	1 (4.2)		25 (100.0)		**<0.0001** ^a^
Grade 1	12 (50.0)		-		
Grade 2	8 (33.3)				
Grade 3	3 (12.5)				
**CTCAE - Creatinine clearance** **(D5) (*n*, %)**					
Grade 0	0		12 (48.0)		**0,0006** ^b^
Grade 1	5 (20.8)		2 (32.0)		
Grade 2	15 (62.5)		1 (8.0)		
Grade 3	2 (8.3)		0		
Grade 4	2 (8.3)		0		
**RIFLE (D5) (*n*, %)**					
AKI not determined by this criterion	0		6 (100.0)		**<0.0001** ^a^
Risk (R)	13 (54.2)		-		
Injury (I)	8 (33.3)				
Failure (F)	3 (12.5)				
**AKIN (D5) (*n*, %)**					
AKI not determined by this criterion	0		6 (100.0)		**<0.0001** ^a^
Grade 1	21 (87.5)		-		
Grade 3	3 (12.5)				

AKI, acute kidney injury; AKIN, acute kidney injury network; CTCAE, Common Terminology Criteria for Adverse Events (assessment according to increase in serum creatinine) (version 4.03); D5, 5th day after the first cycle of cisplatin chemotherapy; *n*, absolute number of patients; RIFLE, risk, Injury, Failure, Loss, and End-stage kidney disease; Scr, serum creatinine; SD, standard deviation.

^a^ Mann-Whitney test.

^b^ Fisher’s exact test.

The statistically significant differences were showed by the bold.

### 3.3 *In-silico* analysis of miRNA potential targets

Urinary miRNAs differentially expressed in sequencing with fold regulation >7.0 or < -7.0 were selected for *in silico* analysis, namely: hsa-miR-6729-5p, hsa-miR-1238-5p, hsa-miR-4706, hsa-miR-6805-5p, hsa-miR-6729-3p, hsa-miR-3928-3p, hsa-miR-4750-5p, hsa-miR-1247-5p, and hsa-miR-4322. A 10th miRNA, hsa-miR-21-5p, was also chosen for the analysis after a literature review (Torso et al., 2021).

As a result of the enrichment analysis, the 50 most statistically significant canonical signaling pathways predicted by the Ingenuity Pathway Analyzes (IPA^®^, Qiagen bioinformatics) are shown in Supplementary Figure S1. The analysis considers the genes predicted by the up- and downregulated urinary miRNAs.

### 3.4 Validation of miRNA candidates for biomarkers of nephrotoxicity

Table 3 lists the miRNAs that revealed differential expression between the two groups in the sequencing, with a fold regulation >5.0 or < -5.0 and a *p*-value <0.05 (the fold change, fold regulation, and *p*-value data analysis were provided by the GeneGlobe software). Five miRNAs were selected for further validation based on three criteria adopted in this study: hsa-miR-1238-5p, hsa-miR-4322, hsa-miR-4706, hsa-miR-6729-5p, and hsa-miR-6805-5p.

**TABLE 3:** Urinary miRNAs differentially expressed between participants with and without cisplatin-induced nephrotoxicity, with a *p*-value <0.05. The miRNAs are organized in the table in descending order according to their fold regulation value; the five miRNAs selected for validation are highlighted in bold.

miRNA	Fold regulation	*p*-value^a^
*Upregulated*		
**hsa-miR-6729-5p**	**23.74**	**<0.0005**
**hsa-miR-1238-5p**	**15.18**	**<0.0005**
**hsa-miR-4706**	**13.61**	**<0.0005**
**hsa-miR-6805-5p**	**13.55**	**<0.0005**
hsa-miR-6729-3p	9.03	<0.005
hsa-miR-3928-3p	7.74	<0.005
hsa-miR-4750-5p	7.46	<0.005
hsa-miR-1247-5p	7.06	<0.005
hsa-miR-6721-5p	6.91	<0.005
hsa-miR-484	6.34	<0.05
hsa-miR-1247-3p	5.48	<0.05
hsa-miR-5698	5.37	<0.05
hsa-miR-425-3p	5.22	<0.05
hsa-miR-203a-3p	5.15	<0.05
*Downregulated*		
hsa-miR-6087	−5.37	<0.05
hsa-miR-615-5p	−5.83	<0.05
**hsa-miR-4322**	**−26.46**	**<0.0005**

^a^ Wald test. Upregulated miRNAs, are the ones most expressed among participants with cisplatin-induced nephrotoxicity, while downregulated miRNAs, are the ones most expressed in the group who did not develop nephrotoxicity.

In accordance with the fold regulation calculation method, the four selected upregulated miRNAs had a higher mean expression (calculated according to the UMI-Unique Molecular Identifier-) in the group with cisplatin-induced nephrotoxicity; similarly, the only selected downregulated miRNA had a higher mean expression in the group without nephrotoxicity (Supplementary Table S1). Because we were looking for a biomarker that could identify patients with nephrotoxicity early, the best scenario would be if the miRNA were more expressed in the group of patients with AKI.

Using the 2^−ΔΔCT^ method, Table 4; Supplementary Figure S1 present the expression of the six urinary miRNAs at baseline and 5 days (D5) after the first treatment cycle, normalized according to the expression of endogenous hsa-miR-875-5p. Although expression variations were found between the two groups for all evaluated miRNAs, only hsa-miR-6805-5p exhibited a statistically significant fold regulation at D5 (FR = −20.1, *p* = 0.0001).

**TABLE 4:** Fold regulation (before and 5 days after the first cisplatin chemotherapy cycle, D5) and variation (D5—baseline) of the six urinary miRNAs chosen for validation, assessed by quantitative Real-Time PCR (RT-qPCR) and normalized according to endogenous hsa-miR-875-5p expression (Torso et al., 2023a).

miRNA	Baseline		D5		Variation (%) (D5 - baseline)		
	Fold regulation	*p*-value*	Fold regulation	*p*-value*	Participants with cisplatin-induced nephrotoxicity (*n* = 24)	Participants without cisplatin-induced nephrotoxicity (*n* = 25)	*p*-value*
**hsa-miR-21-5p**	−1.6	0.4329	−1.7	0.1372	127.7 ± 482.9	14.5 ± 40.9	0.4657
**hsa-miR-1238-5p**	−2.2	0.1554	1.2	0.2465	5.6 ± 23.8	0.7 ± 2.2	0.9882
**hsa-miR-4322**	3.5	0.5618	1.4	0.4242	3.1 ± 9.7	2.0 ± 6.0	0.8005
**hsa-miR-4706**	−5.8	0.6413	35.1	0.3892	56.8 ± 220.1	−0.8 ± 0.4	0.6374
**hsa-miR-6729-5p**	14,840.8	0.3097	8,235.1	0.3886	138.2 ± 650.6	0.5 ± 2.9	0.9775
**hsa-miR-6805-5p**	−1.8	0.4719	−20.1	**<0.0001**	−0.8 ± 0.4	7.0 ± 30.7	**0.0001**

The statistically significant differences were showed by the bold.

Spearman correlation coefficients were calculated to verify the existence of a correlation between miRNA expression with creatinine and creatinine clearance, with a correlation coefficient (R) ≥ 0.5 considered a strong correlation. No significant associations were found for any of the six validated miRNAs (data not shown).

### 3.5 Receiver operating characteristic curve (ROC)

**FIGURE 3:**
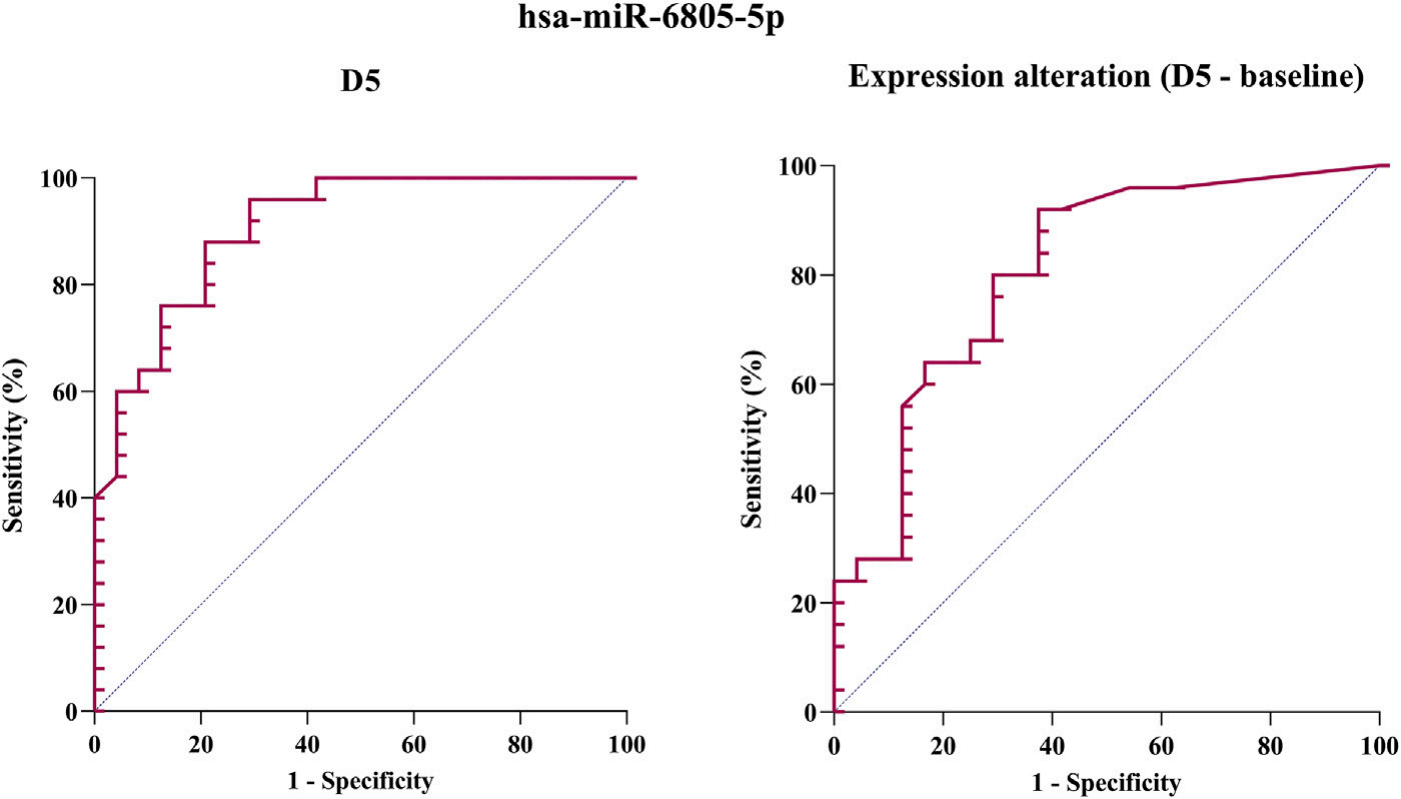
Receiver operating characteristic (ROC) curve of hsa-miR-6805-5p expression 5 days after cisplatin administration (D5) (on the left) and considering the variation in expression between D5 and baseline (on the right).

For hsa-miR-6805-5p (the only miRNA whose expression comparison between the two groups had a *p*-value < 0.001), a receiver operating characteristic (ROC) curve was generated in order to evaluate the miRNA’s ability to distinguish between those with and those without cisplatin-induced nephrotoxicity. Figure 3 presents two ROC curves: one assessing hsa-miR-6805-5p expression 5 days after the first cisplatin chemotherapy cycle and the other considering the individual variation of this expression (D5 - baseline).

Table 5 shows the area under the curve (AUC), cut-off, sensitivity, and specificity for both cases. It is possible to observe that the AUC ranges between 0.8 and 0.9 in both ROC curves, which can be considered desirable for the purposes of monitoring AKI induced by cisplatin. Furthermore, the evaluation of the hsa-miR-6805-5p expression on D5 showed improved specificity (0.880), while the evaluation of the variation in its expression in relation to the baseline period showed greater specificity (0.920). For this reason, in order to improve the diagnosis of AKI, it would be advisable to use these two assessments of hsa-miR-6805-5p together.

**TABLE 5:** Area under the curve (AUC), cutoff, sensitivity, and specificity of hsa-miR-6805-5p expression (both considering 5 days after cisplatin administration, D5, and considering the variation in expression between D5 and baseline) as a biomarker of cisplatin-induced nephrotoxicity.

has-miR-6805-5p	AUC (95% CI)	Optimal cut-off point	Sensitivity	Specificity
**D5**	0.910 (0.832–0.988)	0.0005561586	0.792	0.880
**Variation (D5—baseline)**	0.812 (0.690–0.934)	−0.9619558	0.625	0.920

### 3.6 *In-silico* analysis of hsa-miR-6805-5p

Using the mirPath v.3 software, a total of four predicted target pathways regulated by hsa-miR-6805-5p were found, all with a *p*-value < 0.05: 1) WNT signaling pathway (associated with four genes); 2) cell adhesion molecules (associated with four genes); 3) O-glycan biosynthesis (associated with two genes); and 4) glycosaminoglycan biosynthesis (associated with a gene). As the first two pathways cited presented a greater number of associated genes, they were selected as the object of study.

## 4 Discussion

To the best of our knowledge, this is one of the few studies in humans that aims to investigate the use of miRNAs as potential biomarkers of cisplatin-induced nephrotoxicity (Torso et al., 2021).

Firstly, the study cohort’s epidemiological characteristics were consistent with those previously reported in other studies (Wong et al., 2011; Rettig and D’Souza, 2015; Ribeiro et al., 2017; Johnson et al., 2020): the majority were male who were white, over 50 years old on average, had sustained exposure to tobacco or alcohol, and were diagnosed with locally advanced disease. Among other factors, one possible reason for the disparity in incidence rates between genders is the more frequent smoking and/or alcohol consumption among male, both of which are known to increase the risk of developing HNSCC (Ribeiro et al., 2017; Chow, 2020). As for the late diagnosis, delay or difficulty in accessing specialized services and the devaluation of symptoms are possible causes of this observation.

A statistically significant difference was seen between the two groups of participants for both the traditional renal markers (serum creatinine and creatinine clearance) and the adopted AKI categories (AKIN and RIFLE) used to monitor renal function.

Turning now to the treatment protocol investigated, it is pertinent to note that despite the significant progress in the field of oncological pharmacology, cisplatin remains a widely used chemotherapeutic agent (Manohar and Leung, 2018). Furthermore, since successful chemotherapy is imperative for a favorable outcome in advanced HNSCC, it is mandatory to investigate predictive biomarkers for cisplatin-associated nephrotoxicity.

Among the six miRNAs profiled in the current study as those potential biomarkers, four were upregulated (hsa-miR-6729-5p, hsa-miR-1238-5p, hsa-miR-4706, and hsa-miR-4322) and two were shown to be downregulated (hsa-miR-6805-5p and hsa-miR-21-5p); however, this difference in expression pattern between the groups was only significant for hsa-miR-6805-5p. In this case, the negative fold change result in the validation means that hsa-miR-6805-5p showed a tendency toward greater expression in the group without cis-platin-induced nephrotoxicity compared to the group that had it. It is too early to draw any conclusions, but this miRNA could possibly be nephroprotective, which could explain the lower incidence of nephrotoxicity in the group. Thus, it is suggested that functional studies be conducted with the aim of better understanding the role of this miRNA in AKI.

Additionally, as shown in Supplementary Figure S2, treatment with cisplatin resulted in a significant downregulation of this miRNA in the group that experienced nephrotoxicity. The remaining question is which event is the cause and which is the consequence: whether this decrease is what causes the nephrotoxicity condition or whether it is the fact that these patients, at baseline, had lower expression of this miRNA, which made them more susceptible to nephrotoxicity. This study alone is not able to provide an answer to this question.

The ROC curve of the only miRNA with a *p*-value <0.0001 (hsa-miR-6805-5p) was developed to evaluate its performance as a biomarker, measuring its accuracy in diagnosing AKI (which means to detect or confirm this condition (Califf, 2018)). The fluctuation of its expression compared to the basal period revealed excellent specificity (0.920), while the evaluation of its absolute expression in D5 showed greater sensitivity (0.792). For this reason, the integrated use of these two assessments as AKI diagnosis biomarkers seems to be an interesting approach. However, the exact correlation between hsa-miR-6805-5p and traditional biomarkers (including creatinine and creatinine clearance) must still be elucidated, along with its expression pattern in larger cohorts with other pathological conditions.

To date, the hsa-miR-6805-5p has been scarcely studied; the few studies published indicate its involvement mainly in acute myocardial infarction and Cushing syndrome. In both cases, its expression was found to be downregulated in patients with the disease; however, as this difference was not considered significant, it was not possible to verify its value as a biomarker through the ROC curve. Given that miRNAs are epigenetic markers, individual variability (Weber et al., 2010; Schwarzenbach et al., 2015) may also influence miRNA expression. As their expression profile evaluation is given in a particular state, dynamically modulated miRNAs may be lost, especially when a limited number of samples are evaluated (Weber et al., 2010). Therefore, it is essential to carry out further clinical studies with hsa-miR-6805-5p, not only to make these data more robust but also so that it can be used as an AKI biomarker in clinical contexts other than the one assessed by this study.

Hsa-miR-1238-5p and hsa-miR-4706 were the only two miRNAs among the six validated to exhibit opposing fold regulation results before and after cisplatin treatment (which means upregulation prior to therapy, downregulation following, and *vice versa*). Furthermore, two of the five miRNAs chosen from sequencing, hsa-miR-6805-5p and hsa-miR-4322, had unexpectedly different results from those obtained from quantitative Real-Time PCR (RT-qPCR) (while one technique indicated that the miRNA was upregulated in the D5, the other showed it to be downregulated). As the miRNA expression profile by RT-qPCR is currently considered the standard method for small RNAs (D’haene et al., 2012), these observed data highlight the importance of validation (where the results found by sequencing will not necessarily be the same).

The *in silico* analysis performed with the ten most relevant miRNAs showed numerous genes associated with the regulation of signaling pathways associated with cisplatin-induced nephrotoxicity, including PIK3 (phosphatidylinositol-3-kinase)/AKT and *SIRT2-*mediated mechanisms, both previously associated with cell death and cisplatin-induced kidney injury (Potočnjak and Domitrović, 2016; Jung et al., 2020). The second *in silico* analysis performed was focused essentially on hsa-miR-6805-5p since it showed better performance as a biomarker of nephrotoxicity. This analysis resulted in the selection of signaling pathways to be studied in an effort to associate them with cisplatin-induced nephrotoxicity.

**FIGURE 4:**
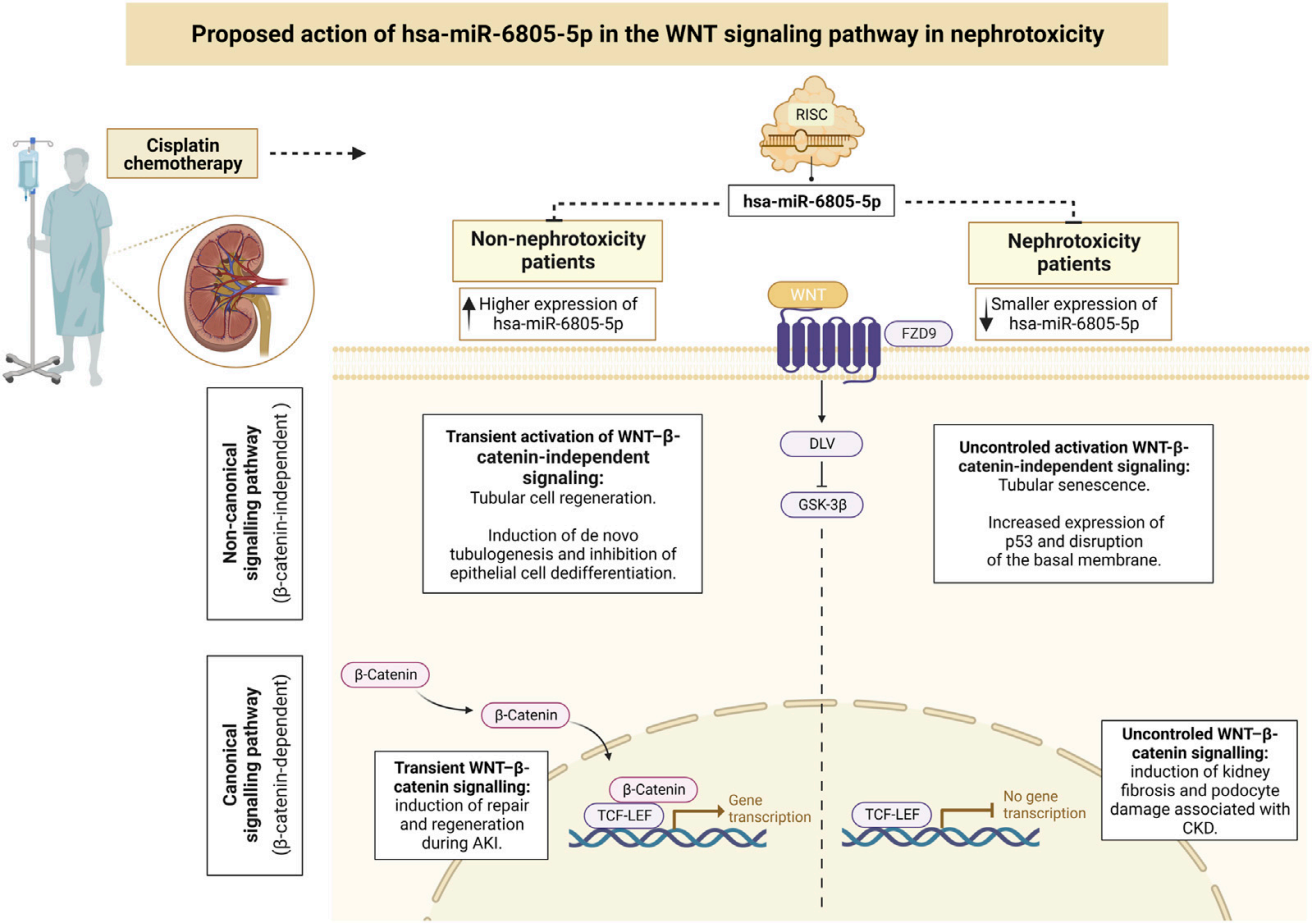
Proposed action of hsa-miR-6805-5p in the WNT signaling pathway in nephrotoxicity. The role of miRNAs in tubular epithelial cells can contribute to the regulation of cell regeneration (where normal repair might restore cell integrity and function) and to apoptotic and fibrotic pathways (being directly implicated in kidney injury) (Mahtal et al., 2022). Created with BioRender.com, accessed on 14 June 2023. AKI, acute kidney injury; CKD, chronic kidney disease; TCF-LEF, T cell factor-lymphoid enhancer factor.

Although the pathological events that underlie AKI repair and fibrosis are not widely recognized, it is already known that disordered repair results in the development of fibrosis, tubular atrophy, and progression to chronic kidney disease (CKD) (Mahtal et al., 2022). Starting with the WNT signaling pathway, it can be both beneficial and harmful in AKI: while transient WNT signaling promotes repair and regeneration, uncontrolled signaling is associated with kidney fibrosis and podocyte damage in CKD. Therefore, an attempt to associate the predicted target of hsa-miR-6805-5p with cisplatin-induced nephrotoxicity was made possible by the *in silico* analysis: the current research proposes that hsa-miR-6805-5p may promote greater and better regulation of the WNT pathway, resulting in preservation of renal function (in other words, induction of repair and regeneration during AKI). This would explain why cisplatin-associated nephrotoxicity is less common in patients with increased expression of this miRNA (Figure 4).

**FIGURE 5:**
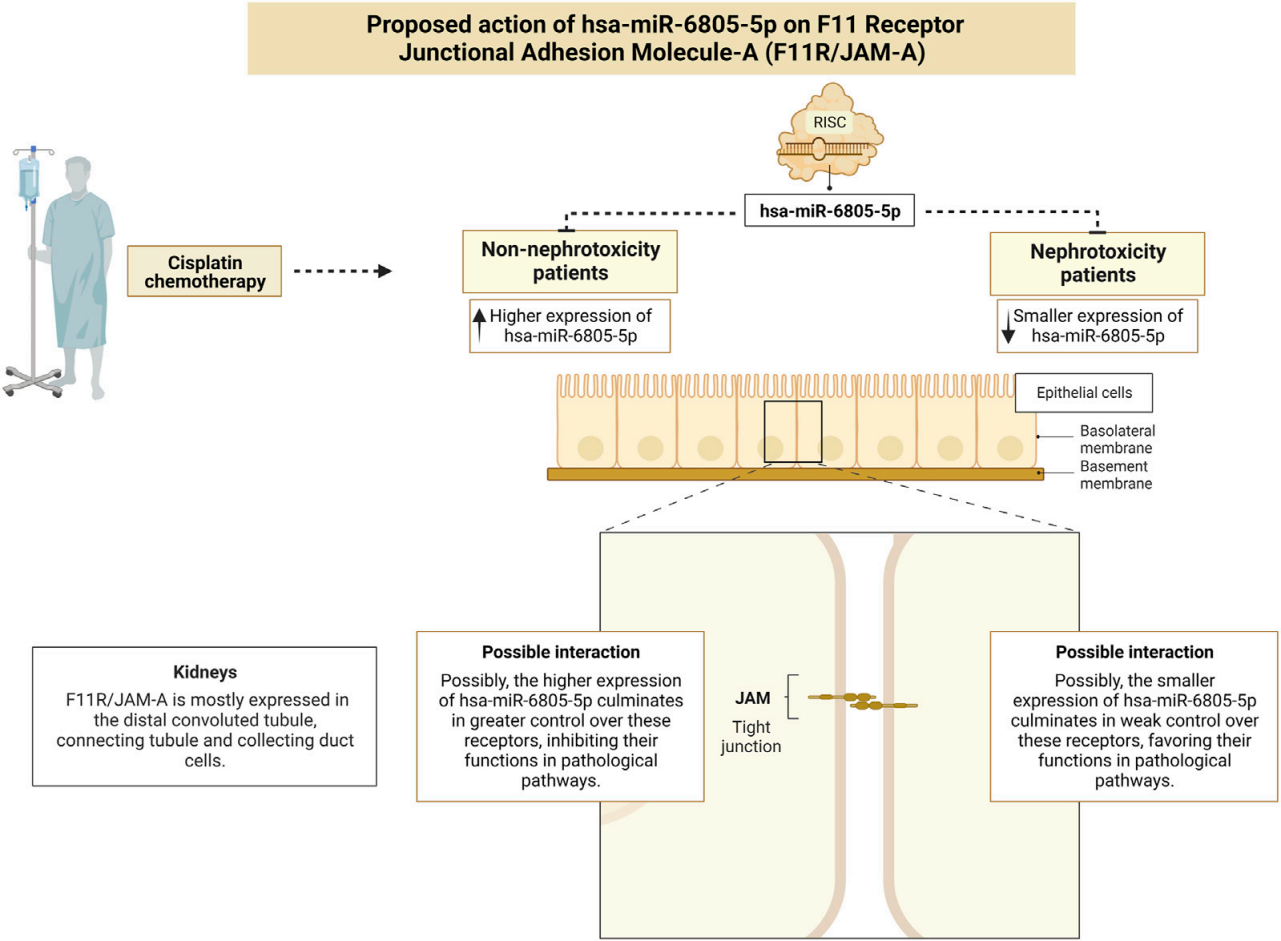
Proposed action of the F11 Receptor - Junctional Adhesion Molecule-A in epithelial cells and its association with nephrotoxicity. Created with BioRender.com, accessed on 14 June 2023.

As for the second pathway predicted for hsa-miR-6805-5p, the cell adhesion molecules, the main target gene was F11 Receptor Junctional Adhesion Molecule-A (F11R/JAM-A). Unlike the WNT pathway, whose role in AKI has already been clarified, the involvement of F11R/JAM-A in this pathogenesis is still unclear. The JAMs are cell adhesion molecules, mainly expressed in epithelial and endothelial cell intercellular junctions, whose regulatory biological role is based on their ability to trigger intracellular cascades of signals at intercellular contact sites (Ebnet, 2017; Czubak-Prowizor et al., 2022; Wang and Liu, 2022). In animal models, the upregulation of JAM-A in different organs, including the kidneys, has been shown to trigger stable elevations in blood pressure, possibly suggesting a pathogenic role for JAM-A in arterial hypertension (Xu et al., 2012). Thus, we propose conducting future *in vitro* and/or *in vivo* studies that verify with greater precision the performance of hsa-miR-6805-5p in these receptors: we believe that, possibly in patients who did not develop nephrotoxicity, the higher expression of hsa-miR-6805-5p culminates in greater control over these receptors, inhibiting their functions in pathological pathways (Figure 5).

Using an *in silico* analysis requires an extra recommendation of caution. As the tools employed in these analyses are reliant on sequence-based prediction to pair miRNAs with target genes, a large set of potential targets is generated, many of which have little functional value. Therefore, it is up to the investigator to determine which ones are associated with their pathways of interest. As a result, the analysis performed by this tool can be relatively partial, and for its results to be reliable, they must be confirmed by *in vitro* and/or *in vivo* studies.

As the last consideration, the advantages and limitations of employing miRNAs as biomarkers deserve a brief discussion. First, the non-invasive attribute of miRNA analysis reduces patient discomfort and enables repeated sampling for long-term monitoring, allowing healthcare providers to make well-informed decisions on patient care (Dobrzycka et al., 2023). Particularly when it comes to urine samples, it is also appropriate for both inpatients and outpatients. In terms of hemolysis, urinary miRNAs have an additional benefit over plasma ones. Due to the rupture of erythrocytes containing miRNAs, hemolysis may influence the concentrations of particular circulating miRNAs in plasma samples (Kirschner et al., 2011). In this instance, hsa-miR-6805-5p would have the advantage of being unaffected by sample hemolysis.

Second, due to the rapid oscillation in their expression, miRNAs are quantifiable indicators that provide early and valuable information for clinical decision-making, enabling most of all, individual timely intervention. The RT-qPCR, regarded as the standard method for miRNA expression profile, has high sensitivity, specificity, and reproducibility (D’haene et al., 2012). Since it requires limited technical expertise and can be automated, it is also a desirable technique for routine testing in clinical laboratories.

Undoubtedly, the relatively high cost of miRNA-related methods and assessment techniques is also a challenge, especially when compared to creatinine, the current standard biomarker. What must be considered is the prospect of using it as a more sensitive and specific biomarker; furthermore, miRNAs have the potential to offer insights into the deregulated signaling pathways in AKI, thus potentially identifying novel therapeutic targets. Understanding miRNA expression patterns, including different time periods, is crucial for the characterization of health and sickness regulation. At the same time, the evolution of techniques for the detection and quantification of miRNAs may lower the associated costs, which would make their clinical application even more feasible.

If new and robust evidence confirms the role of these circulating miRNAs, it is expected that their determination before or during chemotherapy may help the clinician determine the risk/benefit of chemotherapy treatment, in addition to better monitoring and use of preventive strategies for nephrotoxicity.

## 5 Conclusion

Given the intrinsic limitations of the traditional kidney biomarkers, miRNAs have emerged as a non-invasive and easy-to-implement alternative that could assist in the diagnosis of AKI patients. Circulating miRNAs offer distinct characteristics, which makes them interesting for use as non-invasive biomarkers for disease diagnosis and prognosis. In patients with head and neck cancer treated with high doses of cisplatin, it was verified that has-miR-6805-5p is an excellent candidate for a treatment-induced nephrotoxicity biomarker: the fluctuation of its expression compared to the basal period revealed high specificity (0.920), and its absolute expression in D5 showed superior sensitivity (0.792). For this reason, the simultaneous use of these two assessments as AKI diagnosis biomarkers seems to be an interesting option.

The findings of the current study encourage future research involving miRNA hsa-miR-6805-5p, examining its specificity, reproducibility, and robustness in clinical studies involving a larger number of patients, in an effort to better understand its value as a biomarker. Furthermore, conducting functional research on this miRNA could be beneficial to understanding its involvement in AKI.

## Data Availability

The original contributions presented in the study are publicly available. This data can be found here: Universidade Estadual de Campinas (UNICAMP), Reposit\xF3rio de Dados de Pesquisa da Unicamp (REDU), https://redu.unicamp.br/, DOI: 10.25824/redu/XT1BEY.
