# Supplementary material for: miR-6805-5p as a biomarker of cisplatin-induced nephrotoxicity in patients with head and neck cancer
Source: Front Pharmacol. 2023 Nov 28;14:1275238. doi: 10.3389/fphar.2023.1275238 (PMC10713822; doi:10.3389/fphar.2023.1275238)
Supplement: Supplementary file 1 [file DataSheet1.docx]

Supplementary Material

# Supplementary Figures


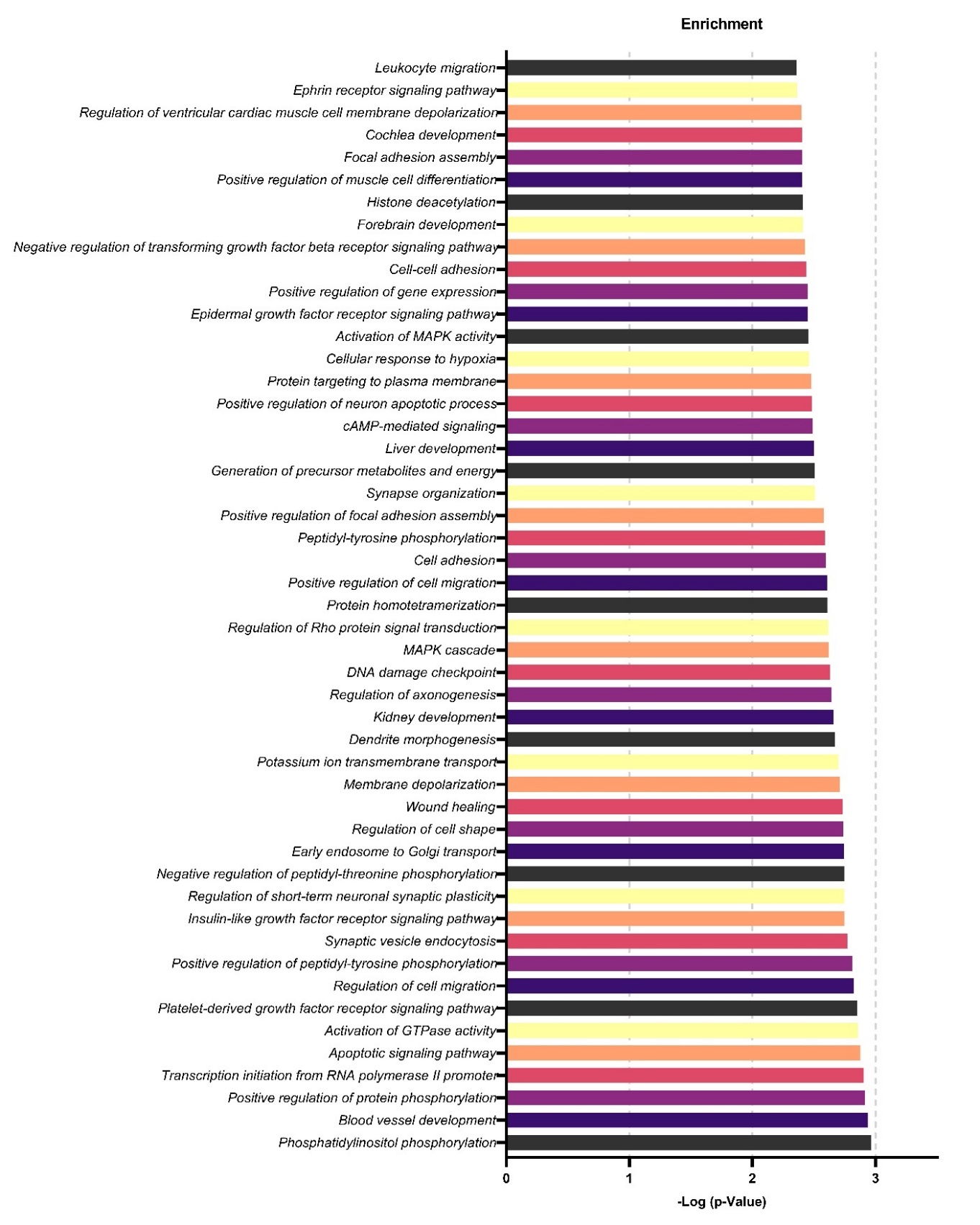


**Supplementary Figure 1.** Enrichment analysis of canonical signaling pathways in which up- and down-regulated miRNA target genes are involved. Enrichment analysis performed by Ingenuity Pathway Analyzes (IPA®, Qiagen bioinformatics), with -log(*p*-value) = 1.3, that is, *p*-value = 0.05 (Fisher's exact test).


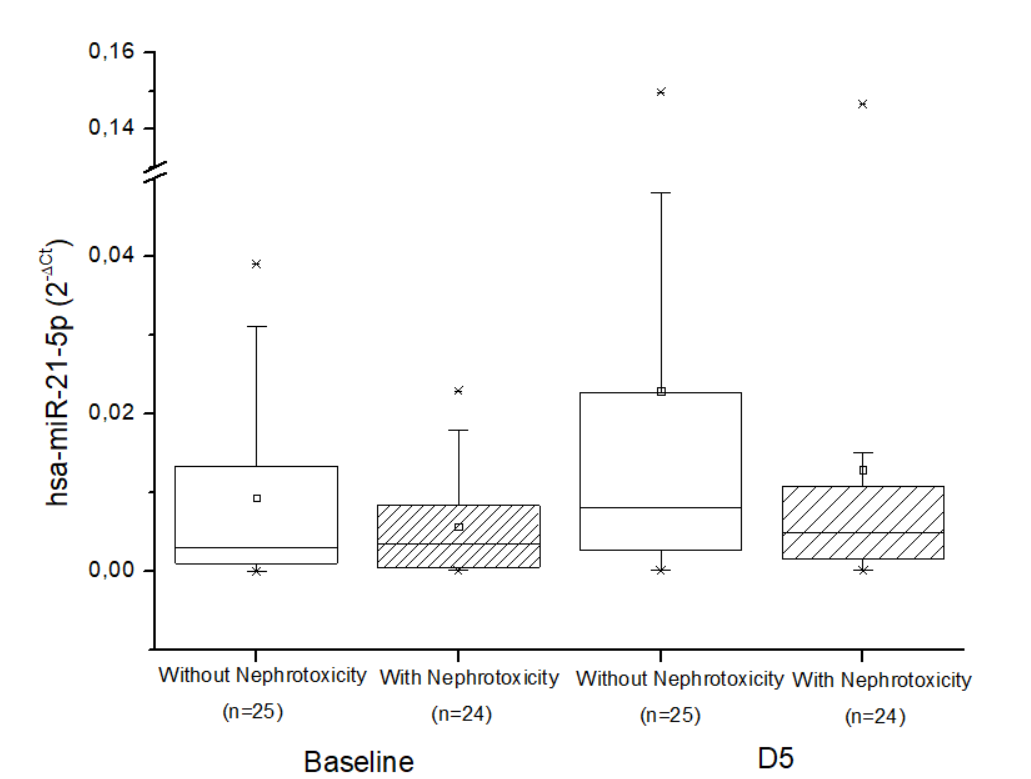

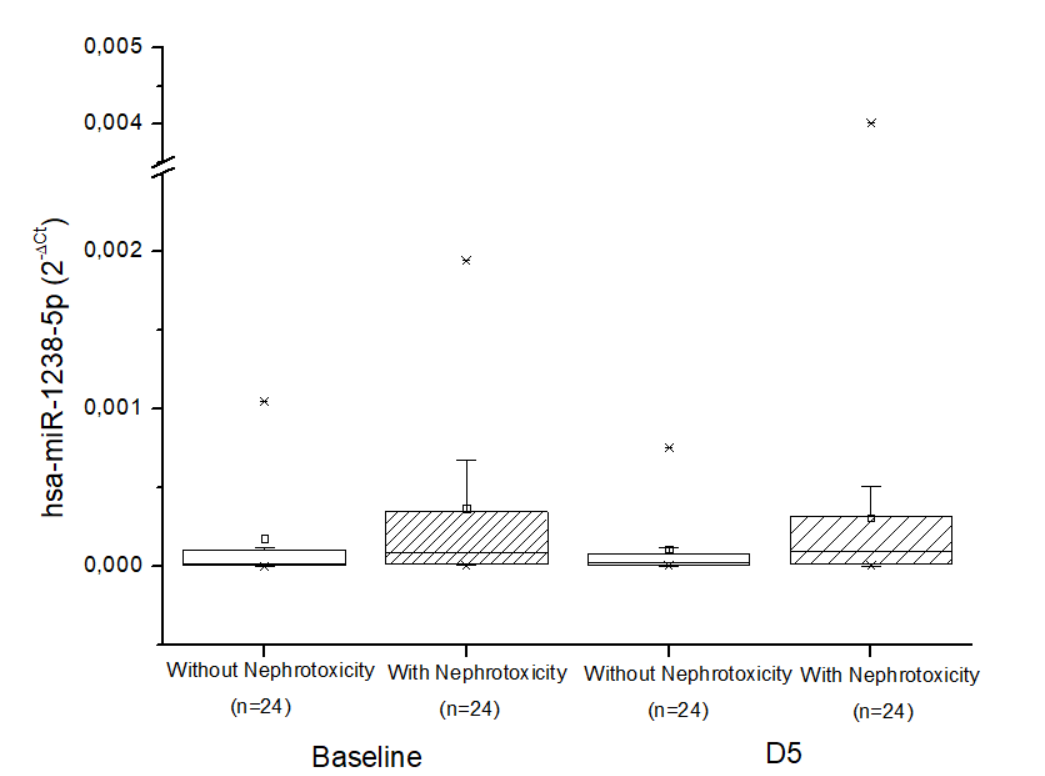

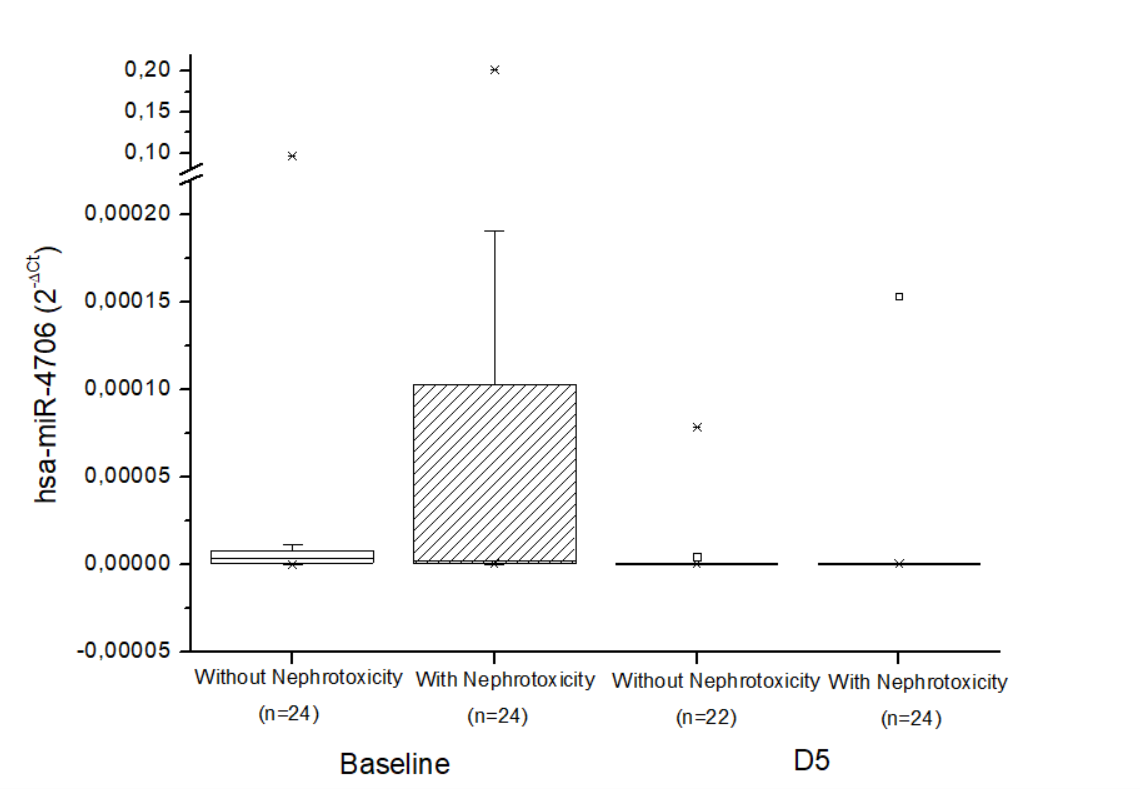


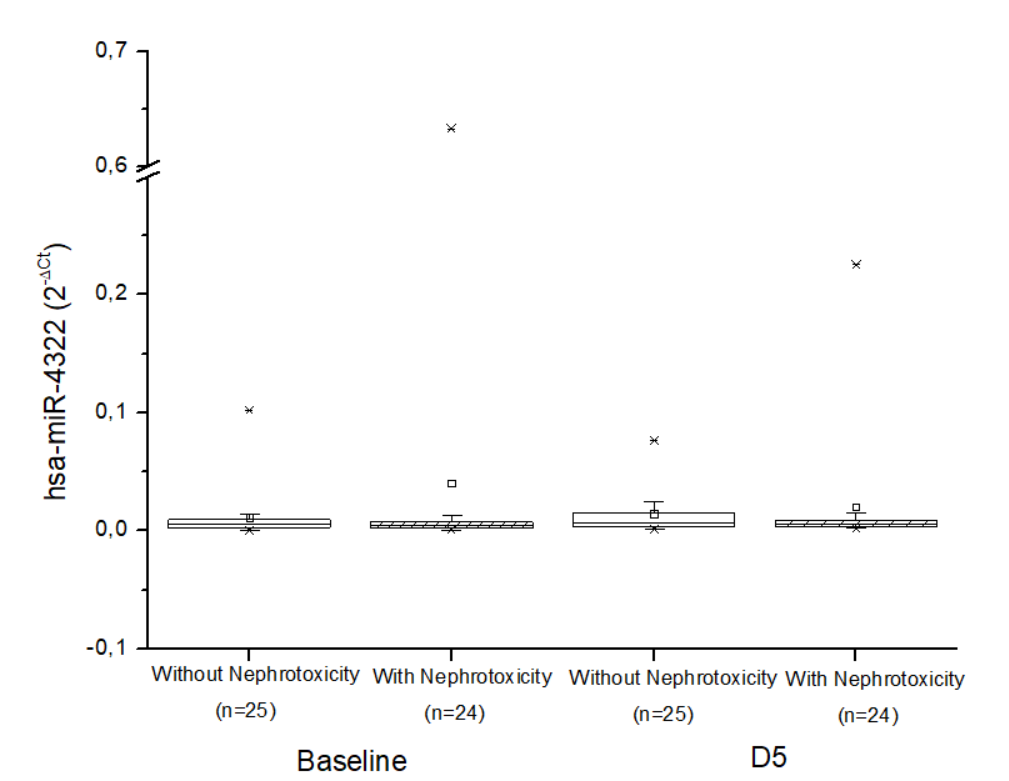


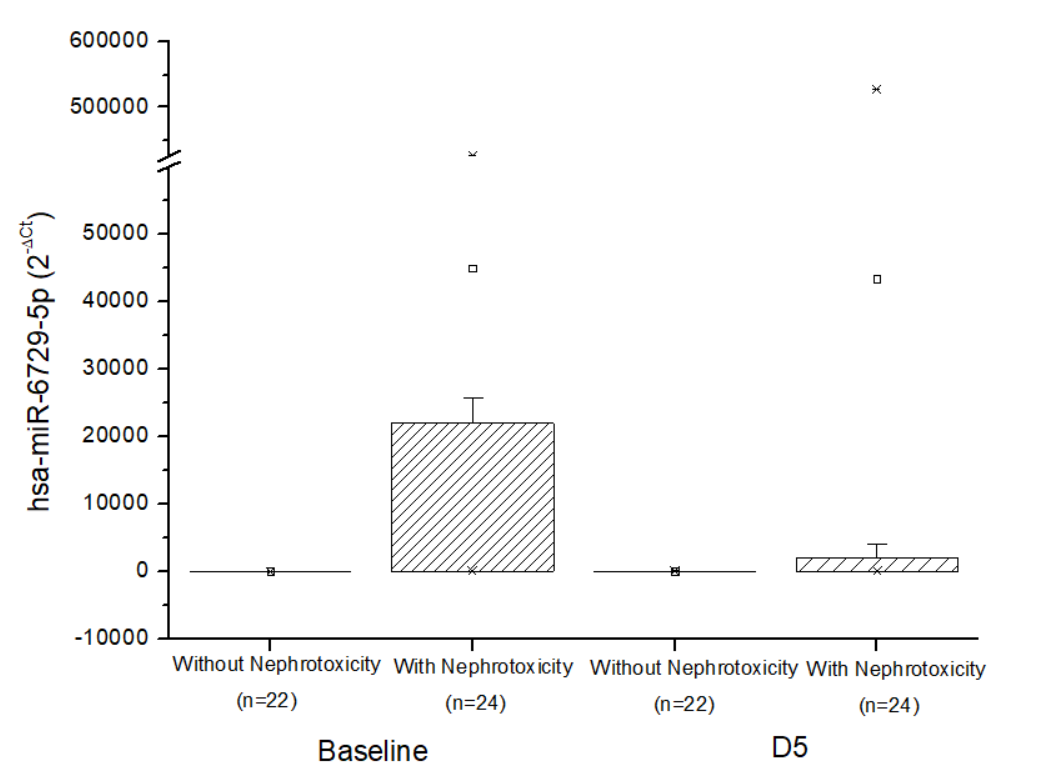

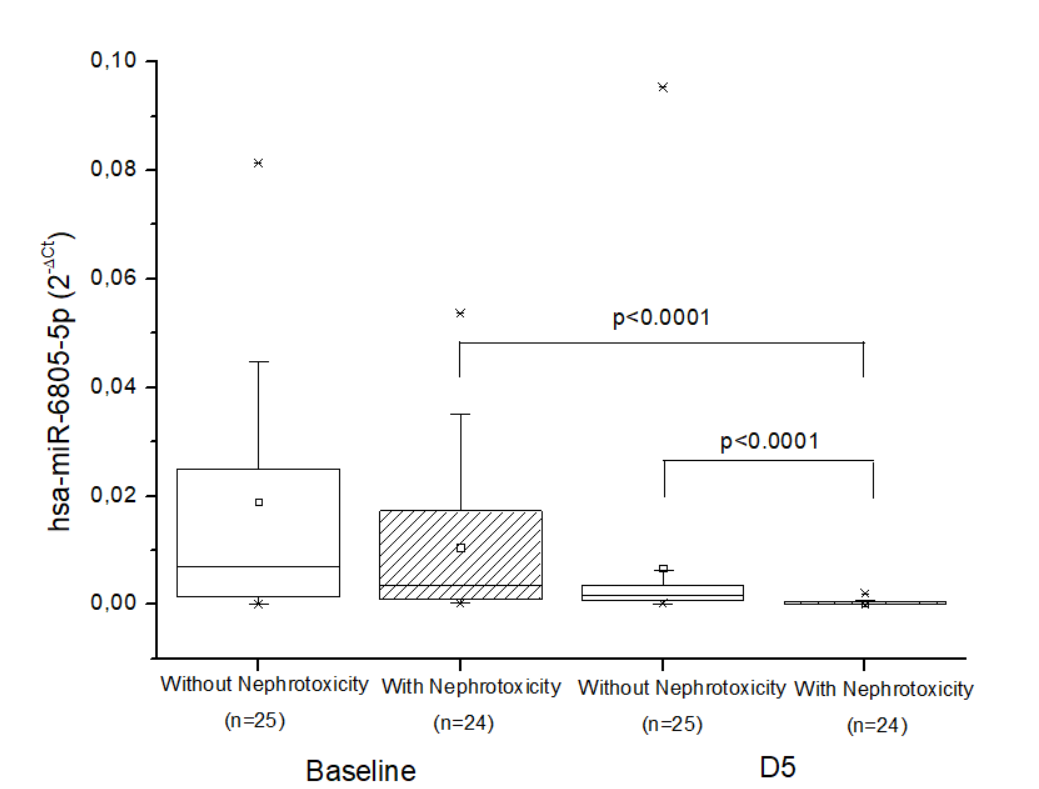


**Supplementary Figure 2.** Expression of hsa-miR-21-5p, hsa-miR-1238-5p, hsa-miR-4322, hsa-miR-4706, hsa-miR-6729-5p and hsa-miR-6805-5p in the nephrotoxicity and non-nephrotoxicity group before and five days after cisplatin administration, assessed by quantitative Real-Time PCR (RT-qPCR) and normalized by endogenous hsa-miR-875-5p expression. D5, 5th day from the first cisplatin chemotherapy cycle.

**Supplementary Table 1.** Details of miRNA expression abundance in the sequencing for the two groups.

| **miRNA** | **Expression**  (mean of UMIs in the group) | |  |
| --- | --- | --- | --- |
|  | **Participants with cisplatin-induced nephrotoxicity (*n* = 6)** | **Participants without cisplatin-induced nephrotoxicity (*n* = 6)** |  |
|  | *Upregulated* | | |
| **hsa-miR-6729-5p** | 44.0 | 3.7 |  |
| **hsa-miR-1238-5p** | 17.5 | 2.5 |  |
| **hsa-miR-4706** | 11.2 | 1.7 |  |
| **hsa-miR-6805-5p** | 19.2 | 2.5 |  |
|  | *Downregulated* | | |
| **hsa-miR-4322** | 4.0 | 202.2 |  |

A, according to the final sequencing analysis by GeneGlobe (Qiagen), “the average UMI -Unique Molecular Identifier- of these miRNAs, and consequently their expression, is relatively low (<10) in the control group or in the case, and is reasonably higher in the other group (>10)”.

**Supplementary Table 2.** Expression of hsa-miR-21-5p, hsa-miR-1238-5p, hsa-miR-4322, hsa-miR-4706, hsa-miR-6729-5p and hsa-miR-6805-5p in the nephrotoxicity and non-nephrotoxicity group before and five days after cisplatin administration, assessed by quantitative Real-Time PCR (RT-qPCR) and normalized by endogenous hsa-miR-875-5p expression.

|  | **2^-∆Ct^ (mean ± SD)** | | | |
| --- | --- | --- | --- | --- |
| **miRNA** | **Participants with cisplatin-induced nephrotoxicity (*n* = 24)** | | **Participants without cisplatin-induced nephrotoxicity (*n* = 25)** | |
|  | **Baseline** | **D5** | **Baseline** | **D5** |
| **has-miR-21-5p** | 0.006 ± 0.006 | 127.671 ± 482.916 | 0.009 ± 0.012 | 14.504 ± 40.901 |
| **hsa-miR-6729-5p** | 44953.374 ±  103097.297 | 43373.870 ±  121706.550 | 3.029 ± 5.224 | 5.267 ± 14.424 |
| **hsa-miR-1238-5p** | 0.000 ± 0.001 | 0.000 ± 0.001 | 0.001 ± 0.003 | 0.000 ± 0.001 |
| **hsa-miR-4706** | 0.011 ± 0.042 | 0.000 ± 0.001 | 0.065 ± 0.299 | 4.371E^-06^ ± 1.683E^-05^ |
| **hsa-miR-6805-5p** | 0.011 ± 0.014 | 0.000 ± 0.001 | 0.019 ± 0.026 | 0.007 ± 0.019 |
| **hsa-miR-4322** | 0.040 ± 0.133 | 0.020 ± 0.049 | 0.011 ± 0.021 | 0.014 ± 0.021 |

D5, 5th day from the first cisplatin chemotherapy cycle.
